# Identification of disease-specific pathways and modifiers in phospholamban R14del cardiomyopathy: rationale, design and baseline characteristics of DECIPHER-PLN cohort

**DOI:** 10.1007/s12471-025-01941-8

**Published:** 2025-03-06

**Authors:** Frederik E. Deiman, Remco de Brouwer, Lukas Baumhove, Nils Bomer, Niels Grote Beverborg, Peter van der Meer

**Affiliations:** Department of Cardiology, University Medical Centre Groningen, Groningen, The Netherlands

**Keywords:** Heart failure, Genetic heart disease, Dilated cardiomyopathy, Phospholamban, Phospholamban R14del, Biomarker discovery

## Abstract

**Background:**

Phospholamban (*PLN*) p.Arg14del (R14del, R14^∆/+^) is the most commonly identified pathogenic variant that causes cardiomyopathy in the Netherlands. Many disease characteristics are still unclear, including the phenotypic triggers, disease progression and disease-specific biomarkers. We aim to gain a better understanding of the R14^∆/+^ pathophysiology by establishing a cohort across the R14^∆/+^ disease spectrum.

**Methods:**

The Disease spECifIc PatHways and modifiERs in PhosphoLambaN r14del cardiomyopathy **(**DECIPHER-PLN) cohort includes 101 participants, categorised as unaffected R14^∆/+^ (*n* = 21), early affected R14^∆/+^ (*n* = 42), end-stage R14^∆/+^ (*n* = 28) and heart failure (HF) of another aetiology (*n* = 10). R14^∆/+^ category was based on left ventricular ejection fraction, HF symptoms, electrocardiogram (ECG) and N‑terminal pro-brain natriuretic peptide concentrations. Of the 91 included R14^∆/+^ carriers, 46 (51%) were female, with a mean age of 55 years (standard deviation: 14). Low-voltage ECG older age, arrhythmias, and conduction and repolarisation abnormalities were common in (early) affected R14^∆/+^ carriers. Serum and plasma were collected from all participants. Induced pluripotent stem cells were generated from fibroblasts of end-stage R14^∆/+^ patients and unaffected R14^∆/+^ family members (*n* = 4) and differentiated into cardiomyocytes. Explanted heart tissue was obtained from R14^∆/+^ patients undergoing cardiac surgery and patients with other HF aetiologies as control. Abnormal PLN protein localisation was confirmed in R14^∆/+^ carriers.

**Conclusion:**

DECIPHER-PLN comprises R14^∆/+^ carriers across the disease and non-disease spectrum and can be used to identify disease-specific biological pathways and modifiers that play a role in R14^∆/+^ cardiomyopathy. Using a multi-omics approach and in vitro disease modelling, we aim to identify novel biomarkers and improve our understanding of R14^∆/+^ pathophysiology. Material is available upon request.

**Supplementary Information:**

The online version of this article (10.1007/s12471-025-01941-8) contains supplementary material, which is available to authorized users.

## Introduction

Cardiomyopathies represent a diverse group of heart muscle disorders characterised by structural and functional abnormalities, often leading to heart failure (HF), arrhythmias and other life-threatening complications [1]. While the genetic basis of familial cardiomyopathies has been extensively explored, the role of specific pathogenic variants, such as p.Arg14del (R14del, R14^∆/+^) in the phospholamban (*PLN*) gene, in disease onset and progression remains an area of active investigation.

PLN is a small protein that is predominantly expressed in the heart and is localised to the sarcoplasmic reticulum (SR) membrane. Here, PLN regulates the reuptake of cytosolic calcium ions into the SR via its interaction with sarcoplasmic reticulum calcium ATPase [2]. R14^∆/+^ is a pathogenic variant of the *PLN* gene where amino acid arginine at position 14 (R14) of the *PLN* coding region is deleted in one allele, causing alterations in protein function [3]. R14^∆/+^ can lead to cardiomyopathy, including dilated cardiomyopathy and arrhythmogenic right ventricular (RV) cardiomyopathy, and is the most common genetic mutation found in cardiomyopathy patients in the Netherlands [4, 5]. To date, over 1500 carriers have been identified, with the majority living in the northern part of the Netherlands [5]. R14^∆/+^ carriers show a wide variety in phenotype. Most R14^∆/+^ carriers present with arrhythmias or subtle electrocardiogram (ECG) changes around the fourth to fifth decade of life, although large differences are seen. In the same family, R14^∆/+^ carriers can present with sudden death or overt HF in their 20 s requiring a heart transplant, or they remain completely asymptomatic until their 70 s. So far, no modifiers (genetic or environmental) have been identified explaining this phenomenon [6]. R14^∆/+^ cardiomyopathy is the predominant reason for heart transplantation or left ventricular assist device (LVAD) implantation at the University Medical Centre Groningen in Groningen, the Netherlands. However, the exact mechanisms by which this mutation contributes to cardiac dysfunction remain incompletely understood.

On a molecular level, R14^∆/+^ is characterised by several hallmarks, including abnormalities in calcium handling, contractile function, cellular metabolism, cellular PLN distribution and sarcoplasmic reticulum dysfunction [7, 8]. Clinically, R14^∆/+^ is primarily characterised by severe cardiac fibrosis, fibrofatty replacement and decreased ECG potentials [4, 9]. The current treatment strategy for R14^∆/+^ includes a standard one-size-fits-all treatment approach that helps modulate symptoms of HF [10] but likely fails to target pathophysiological mechanisms that underlie the disease. In a mouse model of R14^∆/∆^, both eplerenone and metoprolol were insufficient to improve cardiac function and survival [11], indicating the R14^∆/+^ pathophysiology remains poorly understood.

Over the past several years, preclinical studies have identified several promising therapeutic strategies that have successfully improved cardiac function in R14^∆/+^ [12–14]. The translation of these treatment strategies into the clinic will rely heavily on biomarkers, because R14^∆/+^ has a variable disease onset and incomplete penetrance [6]. R14^∆/+^ carriers are likely to benefit most when treatment is initiated at disease onset. Therefore, early detection of disease onset in R14^∆/+^ is of great importance and can be achieved with biomarkers. The heart influences the blood proteome through several factors (e.g. inflammation, coagulation and redox balance) [15]. The identification of blood biomarkers that correlate with the disease state will allow effective monitoring of disease onset and progression and thus more efficient treatment. In the future, targeted therapies for R14^∆/+^ can be initiated upon disease onset, to treat R14^∆/+^ carriers and contribute to improved health and longevity.

In this work, we present the identification of the Disease spECifIc PatHways and modifiERs in PhosphoLambaN r14del cardiomyopathy (DECIPHER-PLN) cohort, which includes participants across the R14^∆/+^ disease spectrum and aims to help identify biomarkers and disease modifiers. For this purpose, clinical data, R14^∆/+^ heart tissue, induced pluripotent stem cells (iPSCs) and blood samples (plasma, serum, peripheral blood mononuclear cells and buffy coats) have been collected that will allow to study the disease onset and pathophysiology of R14^∆/+^ cardiomyopathy.

## Methods

### Study design and main objectives

Understanding the causal biological disease mechanisms enables the development of novel treatment strategies. By harnessing the power of patient-derived material (blood, tissue and iPSCs; see Supplementary Methods in Electronic Supplementary Material) collected in the DECIPHER-PLN cohort, we aim to gain a better understanding of R14^∆/+^ pathophysiology (Tab. 1). Using phosphoproteomics on heart tissue, relevant data will be collected directly from R14^∆/+^ carriers. This will be done in combination with in vitro disease modelling utilising iPSC-derived cardiomyocytes (iPSC-CMs), whereby R14^∆/+^ disease pathophysiology will be studied using a multi-omics approach (phosphoproteomics and proteomics) in combination with extensive functional characterisation (e.g. assays on contractility, calcium handling, metabolism and immunofluorescence) of R14^∆/+^ iPSC lines. Importantly, these iPSC lines will allow us to better understand why one person in a family develops the R14^∆/+^ phenotype and the other does not. iPSC-CMs are also an excellent platform for monitoring the response to promising drugs that may eventually enter clinical trials.

**Table 1:** Overview of DECIPHER-PLN tentative analysis and baseline characteristics of patients with patient-derived R14^∆/+^ iPSC-lines across disease spectrum

**Tentative analysis**		
*Heart samples*	*Frozen tissue, paraffin, cryo- and electron microscopy sections (10 DCM and 10 R14*^*∆/+*^* patients)*	
Multi-omics	Proteomics, phosphoproteomics	
Histology	Histological analyses, immunofluorescence	
*Plasma samples*	*Plasma, serum, PBMCs (21 unaffected, 42 early affected and 28 end-stage R14*^*∆/+*^* patients)*	
Multi-omics	Proteomics (Olink Explore Panel), ELISA, targeted metabolomics and lipidomics	
*Stem cells*	*Patient derived iPSC-lines (4 unaffected and 4 end-stage R14*^*∆/+*^* patients)*	
Multi-omics	Proteomics, phosphoproteomics	
In vitro disease modelling	Assays on contractility, calcium handling, metabolism and immunofluorescence	
Drug intervention	Phospholamban antisense oligonucleotides	
**Baseline characteristics**		
**Variable**	**End-stage R14**^**∆/+**^** (*n*** **=** **4)**	**Unaffected R14**^**∆/+**^** (*n*** **=** **4)**
Age, years	51.25 ± 7.97	60.00 ± 14.97
Male	1 (25)	3 (75)
BMI, kg/m^2^	28.63 ± 5.81	27.39 ± 4.59
Pulse, bpm	74.50 ± 21.98	67.75 ± 21.88
Systolic blood pressure, mm Hg	97.25 ± 4.50	140.25 ± 9.74
Diastolic blood pressure, mm Hg	62.75 ± 6.85	78.00 ± 6.78
*NYHA class*		
– No heart failure	0 (0)	4 (100)
– III	1 (25)	0 (0)
– IV	3 (75)	0 (0)

Data are mean ± standard deviation or *n* (%)

*BMI* body mass index, *DCM* dilated cardiomyopathy, *ELISA* enzyme-linked immunosorbent assay, *iPSC* induced pluripotent stem cell, *PBMC* peripheral blood mononuclear cell, *NYHA* New York Heart Association, *R14*^∆/+^ phospholamban p.Arg14del

We will test the potential of PLN antisense oligonucleotides, which have been shown to be a promising therapeutic agent to combat R14^∆/∆^ cardiomyopathy in a murine model [13]. In addition, using R14^∆/+^ carrier-derived blood samples, we aim to identify biomarkers using metabolomics and the Olink Proteomics Panel (and validate them using other methods, such as enzyme-linked immunosorbent assay) that can distinguish between R14^∆/+^ carriers across the disease spectrum, which will allow early detection of R14^∆/+^ disease progression.

### Study population

Participants were eligible if they were > 18 years of age, had genetic confirmation of their R14^∆/+^ carrier status, were capable of adequate communication and were able to provide informed consent. Participants were categorised into either unaffected, early affected or end-stage disease groups based on LV ejection fraction (LVEF), signs and symptoms of HF, ECG and N‑terminal pro-brain natriuretic peptide (NT-proBNP) levels. The unaffected R14^∆/+^ group had LVEF > 50% and no HF symptoms. The early affected R14^∆/+^ group showed echocardiographic or ECG abnormalities or NT-proBNP > 200 ng/l without symptomatic HF. The end-stage R14^∆/+^ group presented with LVEF < 40%, history of sustained ventricular tachycardias (VTs) or symptomatic HF with NT-proBNP > 200 ng/l. For the collection of skin biopsies, additional inclusion criteria were used. Skin biopsies were collected in pairs of two: a pair had to be an age-matched family member (an older unaffected R14^∆/+^ carrier was acceptable). Exclusion criteria were that R14^∆/+^ carriers could not participate if they had an extensive skin disorder precluding a biopsy from an unaffected skin area, were known to be allergic to local anaesthetics or had any significant cardiovascular risk factor, non-R14^∆/+^ cardiometabolic disease or other known gene mutation. For the collection of heart tissues, participants had to undergo LVAD implantation or receive a heart transplant. Patients with other HF aetiologies served as the control group.

### Ethical statement

The current study is conducted according to the principles of the Declaration of Helsinki (7th revision, October 2013, Fortaleza, Brazil) and in accordance with the Dutch Medical Research Involving Human Subjects Act (*Wet medisch-wetenschappelijk onderzoek met mensen*). The scientific advisory board of the University Medical Centre Groningen provided ethical approval for the collection of the blood samples, skin biopsies and human heart tissues (protocol numbers 2020.326 and 2020.327, UMCG Research Register number 202000351; ABR number NL73976.042.20). DECIPHER-PLN has been registered at ClinicalTrials.gov (identifier NCT04978987).

### Baseline characteristics

A total of 101 subjects were included: 91 R14^∆/+^ carriers and 10 patients with end-stage HF of an aetiology other than R14^∆/+^. Of the 91 R14^∆/+^ carriers, 21 were classified as unaffected, 42 as early affected and 28 as end-stage R14^∆/+^. Baseline characteristics of the DECIPHER-PLN cohort are described in Tab. 2. R14^∆/+^ carriers that advanced across the disease spectrum were older, had higher circulating NT-proBNP and cardiac troponin T levels, had a lower heart rate, systolic and diastolic blood pressure and a higher New York Heart Association (NYHA) class and were more often previously hospitalised for HF. Significantly more devices were used in affected carriers, with 23 of 28 (85%) end-stage R14^∆/+^ carriers having an implantable cardioverter-defibrillator (ICD), with or without a dual-chamber pacemaker and/or cardiac resynchronisation therapy. End-stage and early affected R14^∆/+^ carriers had worse RV and LV functions, more LV dilation and higher estimated RV peak pressures. At 24-hour Holter examination, more premature ventricular contractions (PVCs) and non-VTs were observed in affected subjects, with a median number of PVCs of 3434 (interquartile range: 353–7232) in end-stage R14^∆/+^ carriers and 57% of these subjects showing non-sustained VTs.

**Table 2:** Baseline characteristics of the DECIPHER-PLN cohort

		**R14**^**∆/+**^ **carriers**		
**Variable**	**Unaffected**	**Early affected**	**End-stage**	***P*-value**
**Baseline characteristics (*n*)**	21	42	28	
Age, years	43.76 ± 14.41	58.71 ± 11.69	57.48 ± 12.06	< 0.001
Male	8 (38)	22 (52)	15 (54)	0.49
BMI, kg/m^2^	27.62 ± 5.66	26.04 ± 3.56	26.51 ± 4.25	0.40
Pulse, bpm	79.00 ± 16.73	62.56 ± 7.77	70.65 ± 12.42	< 0.001
Systolic blood pressure, mm Hg	128.38 ± 12.48	125.83 ± 17.70	106.19 ± 16.60	< 0.001
Diastolic blood pressure, mm Hg	78.52 ± 8.36	76.45 ± 10.22	70.15 ± 8.57	0.006
*NYHA class*				< 0.001
– No heart failure	21 (100)	31 (74)	0 (0)	
– I	0 (0)	0 (0)	6 (21)	
– II	0 (0)	11 (26)	9 (32)	
– III	0 (0)	0 (0)	7 (25)	
– IV	0 (0)	0 (0)	6 (21)	
*INTERMACS class*				0.002
– N/A	19 (90)	31 (74)	20 (74)	
– 6	0 (0)	0 (0)	1 (4)	
– 4	0 (0)	0 (0)	0 (0)	
– 3	0 (0)	0 (0)	5 (19)	
– Unknown	2 (10)	11 (26)	1 (4)	
Previous HF hospitalization	0 (0)	2 (5)	10 (37)	< 0.001
ICD	0 (0)	28 (67)	23 (85)	< 0.001
Smoking	7 (35)	7 (17)	6 (25)	0.30
Hypertension	0 (0)	4 (10)	2 (8)	0.35
Hypercholesterolaemia	0 (0)	4 (10)	2 (8)	0.36
Diabetes mellitus	0 (0)	1 (2)	1 (4)	0.68
NT-proBNP, ng/l	40 (12–111)	278 (133–528)	1484 (495–3322)	< 0.001
cTNT, ng/l	6 (4–9)	13 (11–18)	19 (14–34)	< 0.001
**Echocardiography (*n*)**	13	28	22	
LVEF, %	57.47 ± 4.93	44.71 ± 11.69	26.20 ± 11.58	< 0.001
LVEDd, mm	46.67 ± 4.46	51.57 ± 5.29	56.23 ± 6.88	< 0.001
LVEDs, mm	29.73 ± 4.02	38.50 ± 8.28	49.08 ± 7.89	< 0.001
GLS, %	−18.02 ± 3.14	−14.39 ± 3.57	−12.47 ± 4.61	0.023
RV peak pressure, mm Hg	13.23 ± 9.84	21.45 ± 7.42	25.18 ± 10.71	0.11
TAPSE, mm	23.98 ± 4.34	21.10 ± 3.96	16.77 ± 3.98	< 0.001
**Holter (*n*)**	15	20	7	
Number of PVCs	15 (1–131)	1365 (308–2330)	3434 (353–7232)	< 0.001
VTs?	0 (0)	10 (50)	4 (57)	0.003
nsVT?	0 (0)	10 (50)	4 (57)	0.003
sVT?	0 (0)	0 (0)	0 (0)	1.00
**ECG (*n*)**	21	42	28	
*Rhythm*				0.40
– Sinus	21 (100)	39 (93)	25 (93)	
– AF	0 (0)	0 (0)	1 (4)	
– Paced	0 (0)	3 (7)	1 (4)	
Frequency, bpm	74.33 ± 13.10	60.88 ± 7.88	68.22 ± 11.52	< 0.001
PR time, ms	152.76 ± 23.30	171.02 ± 27.32	183.50 ± 46.86	0.010
QRS time, ms	90.29 ± 20.65	111.43 ± 37.09	106.37 ± 40.90	0.085
*QRS voltage*				< 0.001
– < 0.5 mV in limb leads	20 (95)	30 (71)	12 (44)	
– > 0.5 mV	1 (5)	12 (29)	15 (56)	
QTc, ms	426.14 ± 21.06	435.45 ± 37.33	453.70 ± 54.80	0.057
LBBB	1 (5)	2 (5)	0 (0)	0.51
RBBB	1 (5)	11 (26)	5 (19)	0.12
T wave inversion	8 (38)	26 (62)	11 (44)	0.14

Data are mean ± standard deviation, *n* (%) or median (interquartile range)

*AF* atrial fibrillation*, BMI* body mass index*, cTnT* cardiac troponin T*, GLS* global longitudinal strain, *ICD* implantable cardioverter defibrillator, *L/RBBB* left/right bundle branch block, *LVEDd* left ventricular end-diastolic diameter, *LVEDs* left ventricular end-systolic diameter, *LVEF* left ventricular ejection fraction, *nsVT* non-sustained ventricular tachycardia, *NT-proBNP* N-terminal pro-brain natriuretic peptide, *NYHA* New York Heart Association, *PVC* premature ventricular contraction, *RV* right ventricular, *(s)VT* (sustained) ventricular tachycardia, *TAPSE* tricuspid annular plane systolic excursion

### Generation of patient-derived R14^∆/+^ iPSC lines across R14^∆/+^ disease spectrum

**Fig. 1:**
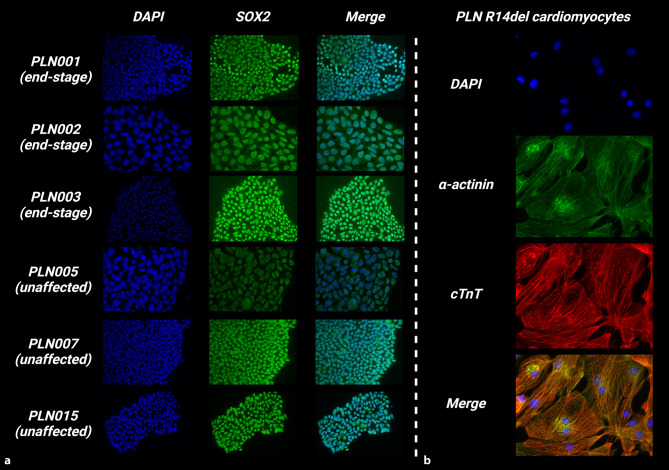
Characterisation and differentiation of induced pluripotent stem cells (*iPSC*) lines from patients with end-stage R14^∆/+^ (rows 1–3) and unaffected family members with R14^∆/+^ (rows 4–6). **a** Immunostaining of 4’,6-diamidino-2-phenylindole (*DAPI*) and pluripotency marker SRY-box transcription factor 2 (SOX2) to detect iPSCs. **b** Immunostaining of cardiomyocyte markers α‑actinin and cardiac troponin T (*cTnT*) to detect iPSC-derived cardiomyocytes

To study R14^∆/+^ disease modifiers, patient-derived skin fibroblasts from end-stage R14^∆/+^ and unaffected age-matched R14^∆/+^ family members were successfully reprogrammed into iPSCs (Tab. 1). To confirm successful reprogramming of fibroblasts into the iPSC lineage, iPSCs were stained for the stem cell marker SRY-box transcription factor 2 (SOX2) (Fig. 1). iPSC generated from fibroblasts expressed SOX2 protein, confirming iPSC line generation. iPSC lines were successfully differentiated into the cardiomyocyte lineage expressing α‑actinin and cardiac troponins (Fig. 1).

### R14^∆/+^ heart tissue harbours fibrosis, fibrofatty infiltrates and abnormal PLN distribution

**Fig. 2:**
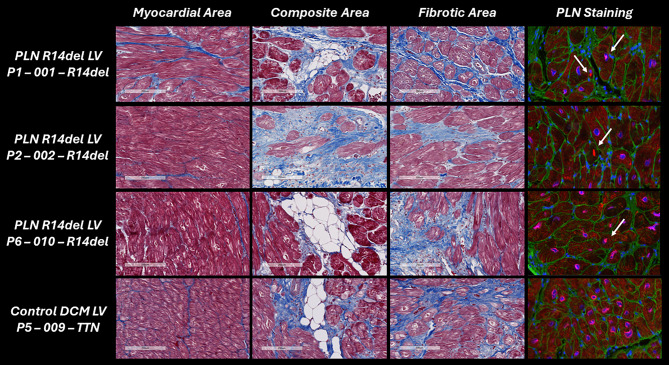
Heart tissue characterisation of patients with R14^∆/+^ cardiomyopathy (rows 1–3) and control dilated cardiomyopathy (*DCM*) tissue (row 4). Masson staining is shown in first 3 columns revealing inter-tissue differences, including myocardial area, composite area and fibrotic area. Last column shows immunostaining of phospholamban (*PLN*) in combination with 4’,6-diamidino-2-phenylindole and wheat germ agglutinin stainings

Using patient-derived tissue allows us to gain a better understanding of disease pathophysiology and identify therapeutic targets. To this end, heart tissues were collected from end-stage R14^∆/+^ patients, with patients with other HF aetiologies as the control group. R14^∆/+^ and control tissues revealed large inter-tissue differences, including regions with seemingly unaffected myocardium, severely fibrotic regions and composite regions consisting of cardiomyocytes, fibrosis and fibrofatty infiltrates (Fig. 2). Immunofluorescent staining of PLN in these tissues revealed abnormal distribution of PLN protein that is specific to R14^∆/+^ and not detected in control HF tissues.

## Discussion

The DECIPHER-PLN cohort has been established and can be used to identify disease-specific biological pathways and modifiers that play a role in R14^∆/+^ cardiomyopathy. Using patient plasma, serum and tissue, we can identify biomarkers that may play a role in R14^∆/+^ cardiomyopathy, with the aim of predicting the onset and progression of R14^∆/+^ cardiomyopathy in R14^∆/+^ carriers. The R14^∆/+^ iPSC lines will be used to determine how iPSC-derived cardiomyocytes from R14^∆/+^ carriers at different ends of the disease spectrum differ in terms of omics and functional characteristics. Our study goal is to identify factors that contribute to R14^∆/+^ disease onset and progression and move towards precision medicine for the treatment of patients with R14^∆/+^ cardiomyopathy.

Genetic cardiomyopathy cohorts are pivotal in advancing our understanding of genetic forms of HF through comprehensive data collection and analysis. The DECIPHER-PLN cohort distinguishes itself from other cohorts that are used to study the pathophysiology of dilated cardiomyopathy leading to HF by the additional collection of R14^∆/+^ carrier-derived tissues through biopsies of the heart and skin, allowing for a direct bedside-to-bench-to-bedside pipeline. Hence, potential new diagnostics, treatments and interventions inspired by clinical observations can be directly tested on human R14^∆/+^ tissues. Promising therapies or diagnostic modalities may then be put to immediate use on the population of interest, leading to quick refinement as well as improved patient outcomes. Large multicentre cohorts such as Heart Failure Molecular Epidemiology for Therapeutic Targets (HERMES) [16], Dilated Cardiomyopathy Consortium (DCC) [17], Sarcomeric Human Cardiomyopathy Registry (SHaRe) [18], the PLN Registry [4, 5] and PHOspholamban RElated CArdiomyopathy intervention STudy (i-PHORECAST) [19] had a larger number of participants and were essential for biomarker discovery. Key biomarkers that have been identified or validated using such cohorts include NT-proBNP, high-sensitivity cardiac troponins, ST2 and galectin‑3 (biomarkers of fibrosis and inflammation), and circulating microRNAs [20]. In this way, we aim to identify a specific biomarker of R14^∆/+^ disease onset and progression. These cohorts were also essential for guiding clinical management and identifying specific genetic risk factors, forming the foundation for cohorts such as DECIPHER-PLN. Similar subject characteristics (e.g. low-voltage ECG and more ICD implantations in the affected R14^∆/+^ group) when compared to other R14^∆/+^ studies and access to a validation cohort for plasma samples obtained during i‑PHORECAST put DECIPHER-PLN in the ideal position to make a push for the development of new therapies and diagnostics for R14^∆/+^ cardiomyopathy.

### Limitations

Although the DECIPHER-PLN cohort is unique in being a cohort of Dutch participants across the disease spectrum of a monogenetic pathogenic variant, there are some limitations. First, blood samples were only collected at one time point, making it difficult to monitor the expression of certain biomarkers over time. In the future, it will be of great interest to follow up on the participants for additional samples, especially those that show R14^∆/+^ disease progression.

Second, the iPSC lines generated from R14^∆/+^ carriers in this cohort do not have isogenic controls. iPSCs are a great platform to study how R14^∆/+^ carriers differ from each other; however, they will not reveal how iPSCs with the exact same genetic background other than R14^∆/+^ are different. Isogenic controls can be achieved using gene editing strategies.

Third, in the DECIPHER-PLN cohort, we were not able to collect heart tissue from unaffected or early affected R14^∆/+^ participants, because they are not severely affected by R14^∆/+^ and there are ethical concerns about collecting heart tissue from R14^∆/+^ carriers who are not undergoing surgery. Fortunately, new initiatives such as the Netherlands Heart Tissue Bank (*Hartenbank*), a biobank that collects heart tissue samples from donors for scientific purposes [21], will be able to provide valuable heart tissue samples in the future.

## Conclusion

The pathophysiology of R14^∆/+^ remains poorly understood, creating an urgent need for more knowledge of R14^∆/+^ and identifying biomarkers that can predict and monitor R14^∆/+^ disease onset and progression. The DECIPHER-PLN cohort has been established to identify biomarkers and gain a better understanding of R14^∆/+^ pathophysiology. We hope that the introduction of our unique cardiomyopathy cohort to the scientific community spreads awareness of the availability of samples and incentivises other scientists to reach out and collaborate or start collecting their own patient samples of HF aetiologies, which will allow us to better understand specific types of HF.

## Supplementary Information

Supplementary methods that describe the collection of plasma, serum and PBMCs, the measurement of Troponin T and NT-proBNP, the generation of iPSCs from skin biopsies, the collection of heart tissue, and the performed histological analysis
