# Supplementary material for: Identification of disease-specific pathways and modifiers in phospholamban R14del cardiomyopathy: rationale, design and baseline characteristics of DECIPHER-PLN cohort
Source: Neth Heart J. 2025 Mar 6;33(4):112–9. doi: 10.1007/s12471-025-01941-8 (PMC11953489; doi:10.1007/s12471-025-01941-8)
Supplement: Supplementary file 1 — Supplementary methods that describe the collection of plasma, serum and PBMCs, the measurement of Troponin T and NT-proBNP, the generation of iPSCs from skin biopsies, the collection of heart tissue, and the performed histological analysis [file 12471_2025_1941_MOESM1_ESM.docx]

**Methods**

**Plasma, serum and PBMCs collection**

Blood samples were collected from all participants. Blood samples were taken through venipuncture. Serum and plasma were derived from the blood samples. Serum was collected by drawing blood into BD Vacutainer Serum Tubes (BD Medical), samples were rested for 30 minutes to allow coagulation, then the tube was spun down at 1500xg for 10 minutes. The upper serum phase was then collected and stored at -80 °C. Plasma was collected by drawing blood into BD Vacutainer EDTA Tubes (BD Medical). Within 30 minutes after blood drawing, the tube was spun down at 2000xg for 10 minutes at room temperature. The upper plasma phase was then collected and stored at -80 °C. Peripheral blood mononuclear cells (PBMCs) were collected by drawing blood into BD Vacutainer CPT tubes (BD Medical) and spinning down at 1600xg for 15 minutes. PBMC phase was transferred and spun down at 300xg for 10 minutes. PBMCs were resuspend and frozen in RPMI supplemented with 40% FCS (Gibco) and 10% DMSO (Sigma) and stored in liquid nitrogen.

**NT-proBNP and Troponin T measurement**

NT-proBNP and Troponin were measured using a Cobas analyzer and the Elecsys NT-proBNP II kit (Roche, #04842464) and Troponin T hs kit (Roche, #05092744).

**iPSC Generation**

Skin biopsies for iPSC generation were collected from end-stage R14^Δ/+^ patients and R14^Δ/+^ family members. Skin biopsies were obtained from end-stage R14^Δ/+^ patients and unaffected R14^Δ/+^ family members. Skin biopsies were collected in phosphate buffered saline (PBS) (Lonza) under sterile conditions from the inner side of the upper arm under local injected anesthesia. Skin biopsies were cut into small pieces and fibroblasts were brought into culture and expanded in AmnioMAX medium (Thermofisher) diluted with Ham’s F12 (Thermofisher) supplemented with 10% FCS (Gibco), 1% GlutaMAX (Gibco) and 1% Pen/Strep (Thermofisher). Fibroblasts were reprogrammed into iPSC using Sendai virus reprogramming following standard procedures[23]. iPSCs derived from skin fibroblasts were stained for stemness using anti-SOX2 (Cell Signaling, #4900), with ALEXA fluor 488 (Invitrogen, A21202). iPSC-CMs were differentiated towards the cardiomyocyte lineage following standard procedures[24], and stained for cardiac markers using anti-cTNT (Abcam, ab45932) and anti-α-actinin (Sigma, A7732), with ALEXA 488 donkey-anti-mouse (Invitrogen, A21202) and ALEXA 488 donkey-anti-rabbit (Invitrogen, A31572).

**Heart tissue collection**

Heart tissue was collected from end-stage R14^Δ/+^ patients undergoing LVAD implantation or heart transplantation, with other etiologies of HF as controls. From participants undergoing LVAD implantation, the heart was derived from the hole that is made for the cannula of the LVAD in the left ventricular free wall. From participants undergoing heart transplantation, tissue from the ventricles and atria were collected after explanting of the heart. Heart tissue was prepared for extensive characterization by storing at -80°C for molecular analysis and as cryo- and paraffin sections for immune(histo)chemistry, and in 2% paraformaldehyde with 2% glutaraldehyde fixative for electron microscopy.

**Histological analysis**

Formalin-fixed paraffin-embedded heart tissue from end-stage patients with R14^Δ/+^ and other etiologies of HF were stained for fibrosis and PLN protein. For assessing fibrosis, slides were stained with Masson’s trichrome stain following standard procedures. Masson’s were imaged using a NanoZoomer 2.0-HT digital slide scanner (Hamamatsu Photonics). To determine PLN protein aggregation, Immunofluorescent staining for PLN was performed using anti-PLN (Cell signaling, 14562S), With Alexa Fluor 555 donkey anti-rabbit IgG (Thermofisher, A31572) as was utilized in combination with wheat germ agglutinin (WGA; Sigma) for staining of the extracellular matrix. Nuclei were stained with 4’,6-diamidino-2-phenylindole (DAPI; Vector Laboratories).

**References**

[23] N. Fusaki, H. Ban, A. Nishiyama, K. Saeki, and M. Hasegawa, ‘Efficient induction of transgene-free human pluripotent stem cells using a vector based on Sendai virus, an RNA virus that does not integrate into the host genome’, *Proc Jpn Acad Ser B Phys Biol Sci*, vol. 85, no. 8, pp. 348–362, Oct. 2009, doi: 10.2183/pjab.85.348.

[24] P. W. Burridge, A. Holmström, and J. C. Wu, ‘Chemically Defined Culture and Cardiomyocyte Differentiation of Human Pluripotent Stem Cells’, *Current protocols in human genetics / editorial board, Jonathan L. Haines ... [et al.]*, vol. 87, no. 1, p. 21.3.1, Oct. 2015, doi: 10.1002/0471142905.HG2103S87.
